# Psychological Pain Measurement in the Context of Suicidal Behavior: Rasch Analysis of the Spanish Psychache Scale Version

**DOI:** 10.3390/jcm14248847

**Published:** 2025-12-14

**Authors:** Jorge L. Ordóñez-Carrasco, Claudia Suárez-Yera, María Sánchez-Castelló, Antonio J. Rojas-Tejada

**Affiliations:** 1 Department of Psychology and Sociology, Faculty of Social and Human Sciences, University of Zaragoza, Calle Ciudad Escolar, 4, 44003 Teruel, Spain; 2 Department of Psychology, Faculty of Psychology, University of Almería, Carretera Sacramento s/n, 04120 Almería, Spain; csy610@ual.es (C.S.-Y.); msc943@ual.es (M.S.-C.); arojas@ual.es (A.J.R.-T.)

**Keywords:** psychological pain, young adults, psychache scale, Rasch model, rating scale model

## Abstract

**Background**: The Psychache Scale (PS) is the most widely used scale to measure psychological pain due to its ease of application, favorable evidence of predictive validity, and adequate psychometric properties from the CTT (Classical Test Theory) approach. This paper aims to contribute to the improvement of the Spanish version of the PS by analyzing its psychometric properties using a Rasch model. **Methods**: Using quota sampling, 905 young adults completed an online questionnaire with the PS. **Results**: The items and response categories showed an acceptable fit to the model and good performance. The separation index indicated three strata for persons. The item-person map showed that persons were placed lower on the psychological pain continuum than item, and some item pairs presented small difference in their severity. The study of men-women DIF (differential item functioning) showed a slight differential functioning only for item 6. **Conclusions**: This study provides new evidence that supports the use of the PS to measure psychological pain.

## 1. Psychological Pain Measurement in the Context of Suicidal Behavior: Rasch Analysis of the Spanish Psychache Scale Version

Suicidal behavior represents a critical public health issue at the international level. According to the World Health Organization, suicide causes approximately 703,000 deaths yearly. That is, 1.3% of all deaths worldwide [1]. However, the study of this topic is complex, mainly due to the concatenation of factors of different nature (e.g., biological, psychological, sociocultural, environmental) that shape and influence this type of behavior [2]. Although numerous factors have shown their influence on the development of suicidal behavior, a large body of theoretical and empirical research highlights the relevant role of psychological pain in the understanding, evaluation, and prediction of this phenomenon, even performing better than classical risk factors such as depression and hopelessness [3,4,5].

Psychological pain (also called psychache, mental pain, psychic pain, emotional pain, or suffering [6]) was originally defined by Shneidman [7] as “the hurt, anguish, soreness, aching, psychological pain in the psyche, the mind”. It is an unpleasant, enduring, and unsustainable feeling characterized by a perception of inadequacy or deficiency of the self [8], a state of intense emotional disturbance and distress that precedes most suicidal behaviors [7,9]. A considerable number of studies have tested the hypothesis of psychological pain as a mediator between different risk factors (e.g., depression, hopelessness, or general distress) and suicidal behavior [10,11,12,13]. Results suggest that all psychological factors were related to suicidal behavior to the extent that they are related to psychological pain. Likewise, some findings reflect an increased suicidal risk due to the interaction between psychological pain and some environmental factors, including exposure to the suicide of someone nearby [14,15]. In summary, psychological pain is a central construct in the study of suicidal behavior, with a robust association with different indicators of suicidal risk [3], as well as a notable contribution to the appearance and development of suicidal behaviors in people with and without a diagnosis of psychopathology [5].

Currently, there are several measurement instruments to assess the severity of psychological pain, for example, the Orbach & Mikulincer Mental Pain Scale (OMMP; [16]), the Mee–Bunney Psychological Pain Assessment Scale (MBPPAS; [17]), and the Psychache Scale (PS; [18]). By virtue of its simple administration and validity evidence regarding its predictive ability, the PS is the most widely used measure to assess psychological pain in the suicidology field [19]. Based on Shneidman’s definition [7] of psychache, the PS has been applied in both clinical and general populations [20,21]. Likewise, this scale has been adapted to different cultural contexts, such as Chinese [22], Polish [23], Portuguese [19], and Spanish [21].

The analysis of psychometric properties has shown that PS scores present high reliability estimates in terms of internal consistency (≥0.90) in different samples (e.g., prisoners, adults, or college students with elevated suicidal risk [24,25,26]). Also, in terms of internal structure, most studies have found a unidimensional solution [27]. However, the fact that the last four items have different response alternatives than the rest of the items has sometimes originated a two-factor structure, although this solution lacks interpretability concerning content and does not indicate a significant contribution to validity compared to the unidimensional structure [28]. In addition to distinguishing between people who attempt suicide and those who do not [18], PS scores were moderately highly associated with depression, hopelessness, and suicidal ideation [26]. Moreover, it is noteworthy that the PS total score better predicted suicidal ideation and suicide attempts (and the number of these) than measures of depression and hopelessness in a sample of college students [29]. A longitudinal (two-year) study by Troister and Holden [13] showed a similar result: PS score was the only predictor of suicidal ideation at baseline, at follow-up, and when examining changes over time in a sample of college students at high suicidal risk.

Although analyses of the psychometric properties of the PS have shown favorable results, previous studies have exclusively adopted the Classical Test Theory (CTT), relying on raw sum scores to quantify the level of psychache. As an advantageous alternative, the main objective of this work is to explore the scores of the Spanish version of the PS by applying a polytomous Rasch model (Rating Scale Model; [30]), which extends the standard dichotomous model [31] to items with graduated response scales. From a measurement perspective, Rasch models allow the calibration of persons and items on a common linear continuum through a probabilistic estimation process [32,33]. Crucially, this overcomes a central limitation of CTT, which implicitly assume unit-weighting for all items regardless of their severity; the Rasch framework locates each item at the specific point on the continuum where it represents a distinct level of pain intensity [34]. Moreover, Rasch analysis entails a prescriptive approach that requires explicit evaluation of key measurement assumptions—such as unidimensionality, local independence, and the monotonic ordering of response thresholds [35]. This provides a methodological scrutiny of whether the scale operates consistently across the continuum of the construct and whether respondents utilize the response categories as intended [36,37].

Capitalizing on these analytical strengths, this study implements statistical procedures unavailable in CTT to provide a more granular analysis of how the variable is measured [38,39]. Specifically, applying this model, and if the data fit the model, we will establish an item-person map to locate the PS items (according to their contribution to the hypothesized continuum of psychological pain) and the persons (according to the level of attributable psychological pain) jointly [40]. Likewise, using this Rasch model, we will assess the adequacy of the response options through the category probability curves [41] and the test accuracy along the continuum through the Test Information Function (TIF; [42]).

Finally, remarkable differences are observed in the prevalence of suicidal behaviors according to sex, being more common suicide in men but more prevalent in other types of suicidal behaviors (e.g., suicidal ideation and suicide attempt) in women [43,44]. Discerning the reasons for these differences involves the study of variables related to suicidal behavior and how they impact men and women to different degrees. Therefore, it is desirable to have a measure of psychological pain that does not generate differences in its scores attributable to issues other than the participants’ ability, regardless of their sex. To examine whether men and women with the same level (or severity) of psychological pain differ in the scoring of each of the PS items (which could be considered a bias that adulterates the interpretation of the scores and, therefore, a source of invalidity), differential item functioning (DIF) will be explored. An item shows biased functioning or DIF when people with a given construct level, who are expected to score similarly on the item, score differently on this item according to a grouping variable [45,46].

## 2. Materials and Methods

### 2.1. Participants

The sample consisted of 905 young Spanish adults recruited from the general population (*M_age_* = 25.91; *SD_age_* = 5.09; range: 18–35 years old; 51.3% female), who participated in an online survey. Regarding sample size sufficiency, for item calibration using polytomous Rasch models, a sample of 500 participants is considered sufficient for most purposes to obtain stable estimates, even under adverse circumstances [47,48]. The type of sampling was by age (33% were 18–23; 33% were 24–29, and 33% were 29–35 years old), sex (50%), and educational level (between 33% and 50% university graduates) quotas, mirroring the composition of the Spanish young adult population. In this sample, 79.6% of young adults did not report suicidal ideation (item 9 of the BDI-II) or other suicidal behavior. The socio-demographic characteristics are shown in Table 1.

### 2.2. Instruments

This study is part of a larger research project in which other instruments (e.g., Beck Hopelessness Scale, Beck Depression Inventory) were administered during data collection. However, only the PS data were analyzed in the present study.

*The Spanish version of the Psychache Scale* (PS; [21]; original version by Holden et al. [18]). Self-administered scale to assess the intensity/frequency of psychological pain consists of 13 items (e.g., *Mi dolor psicológico afecta a todo lo que hago* [*My psychological pain affects everything I do*]). The response options of the Spanish version of the PS are 5-point Likert-type. From item 1 to item 9, the response options range from 1 “never” to 5 “always”, and from item 10 to item 13, the response options range from 1 “strongly disagree” to 5 “strongly agree”. A higher score is interpreted as a higher frequency/intensity of psychological pain. In this sample, scores on the Spanish version of the PS exhibited an excellent estimated reliability coefficient (α = 0.96).

*Socio-demographic variables*. Participants indicated their sex, age, completed education level, work activity, and marital status.

### 2.3. Procedure

The collaborators (i.e., psychology students and research team members) recruited participants from their contacts and social networks, following pre-established quotas. They briefly explained the conditions of the study and provided a link to the online questionnaire. LimeSurvey (web survey platform, http://www.limesurvey.org/, accessed on 1 December 2025) was used to conduct the online questionnaire. Neither collaborators nor participants received any financial incentive for their participation. Before the administration of the questionnaire, participants gave their online consent after being informed of the objectives of the research, the mechanisms that guarantee their anonymity, the data protection law, and the voluntary nature of their participation. This research was approved by the Bioethics Committee on Human Research of the university of the researchers.

### 2.4. Data Analysis

The rating scale model was applied to the 13 items of the Spanish version of PS. This model transforms the raw scores of items and persons into an interval scale with the same metric (with mean 0 and standard deviation 1), referred to as log-odds or logit units [49]. To test whether the empirical data fit the model predictions, the mean square fit statistics (i.e., mean square residual—MNSQ–and standardized mean square residual–ZSTD) were calculated using two indices: infit (inlier-pattern-sensitive fit statistic) and outfit (outlier-sensitive fit statistic). MNSQ values between 0.5 and 1.5 and ZSTD values between −2.0 and 2.0 are considered an acceptable fit [50]. However, a transformation of the ZSTD fit statistic was performed because the null hypothesis of ZSTD (i.e., these data fit the Rasch model exactly) is difficult not to reject when we have a large sample size, which favors a notable degree of misfit to the model. This transformation (ZEMP) allows us to test whether the data are acceptable according to their empirical distribution rather than the theoretical distribution of the model, which makes the interpretation of the fit statistics more useful. To do this, ZSTD was empirically re-standardized to match a unit-normal (Z) distribution to obtain a transformed standard deviation of 1 by dividing ZSTD by the local standard deviation [51]. This local-rescaling reports how unlikely is the amount of misfit to appear if the ZSTD conformed to a random normal distribution with the same variance as the original standardized fit statistics.

Once the fit of the data to the model has been verified, separation indices and strata were estimated for persons and item scores (strata = [4*separation + 1]/3). Both indices are estimates of the sample’s spread relative to the precision (Standard Error) of those measurements [52], and they inform us about the number of statistically different performance levels that the test can identify in the sample: for persons, sensitive to distinguish between high and low performers; for items, verify the order for locating the items on the continuum (i.e., item hierarchy). If the sample is large and normally distributed, then use separation indices rather than Strata [53]. For persons, high separation is considered when the value of the separation index is greater than 2. In the case of the items, a separation index value greater than 3 means there are enough persons to confirm the order of the items (item hierarchy), and the scale includes at least items, with three levels of severity: low, medium, and high [51]. In addition, reliability for persons and items was reported, which in this model refers to the reproducibility of the order in which persons and items are located along the psychological pain continuum [51]. In the case of persons, reliability is considered high when the value is above 0.80. For items, a good reliability value is considered when it is above 0.90 [51]. The degree of precision of the measurement at each level of the continuum was also analyzed graphically using the Test Information Function (TIF; [42]). TIF is usually presented as a graph and shows the amount of information (*y*-axis) for a test at each point along the latent variable (in logits on the *x*-axis; [53]).

Subsequently, the category probability curves [36] were examined. These identify the likelihood of a response being made in any of the possible response categories (i.e., the Likert-type response options of the scale items). Each category is expected to be more likely at some point in the construct. Also, to assess the adequacy of the response categories, thresholds parameters (i.e., the intersection between two contiguous response alternatives where both responses are equally likely) will be analyzed, expecting to find an order consistent with the meaning of the response options (e.g., lower values for the “never-sometimes” threshold than for the “sometimes-often” threshold).

Item-person map was obtained to locate persons according to their psychological pain level or severity, and items according to their contribution to this psychological continuum or difficulty (the logit range of item severities indicates the width of the PS that is covered by the test). Both persons and items are measured on the same scale (logit units), and this makes it possible to compare them along the continuum. The lower part of the map corresponds to the lowest estimates of the construct being measured. The upper part refers to the highest estimates.

Finally, to ensure that all items have similar functioning between men and women with the same level of psychological pain, the differential item functioning (DIF) was examined. To estimate the DIF, two criteria will be considered [51]: (a) the difference in logits between the women subgroup and men subgroup has a size greater than 0.5 logits (i.e., DIF contrast), and (b) the Rasch–Welch *t*-test is statistically significant (*p* ≤ 0.05).

**All analyses were performed using Winsteps version 4.5.0 software (John M. Linacre, Beaverton, OR, USA) [54]** **. Additionally, the item-person map was illustrated using jMetrik version 4.1.1 (Psychomeasurement Systems, LLC, Charlottesville, VA, USA).**

## 3. Results

Regarding the fit analyses, overall, the verification of the fit of the data showed adequate indices for persons and items (Table 2). In more detail, all items exhibited infit indices with values between 0.66 and 1.37 for MNSQ and between −1.51 and 1.28 for ZEMP, except for item 6 “No entiendo por qué sufro” [“I can’t understand why I suffer”] (MNSQ = 1.72; ZEMP = 2.32), which revealed a slight misfit. In the case of the outfit indices, the MNSQ values ranged from 0.75 to 1.35, and the ZEMP values ranged from −1.05 to 1.11, except for item 6 (MNSQ = 1.79; ZEMP = 2.43). In addition, the 13 items comprising the PS covered a range of the psychological pain continuum from −0.95 logits (item 1, less psychological pain) to 0.83 logits (item 11, more psychological pain). Likewise, item-total correlations for the 13 items varied between 0.70 and 0.86.

**Figure 1:**
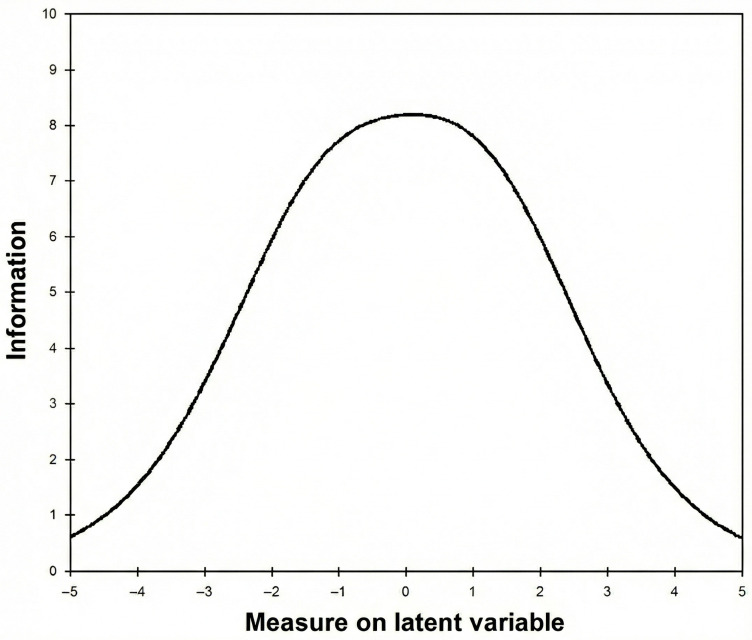
Test Information Function.

The separation indices for persons and items were 2.04 and 8.72, respectively. Due to the floor-ceiling effect of the PS scores in our sample, strata are used to assess how many levels of the PS could be identified. Using the separation index, 3.05 strata of the PS were identified for persons. It is just at the lower limit to be considered acceptable. The reliability for persons was 0.90, suggesting a good replicability of their location according to the psychological level. For items, 11.96 strata were identified. Moreover, the reliability for the items was 0.99, which means a high replicability of the order of the items across the psychological pain continuum. Likewise, the Test Information Function (Figure 1) shows us the accuracy curve where a higher test accuracy is observed in the intermediate values of the psychological pain continuum compared to the extremes of the continuum, where a higher error is expected.

**Figure 2:**
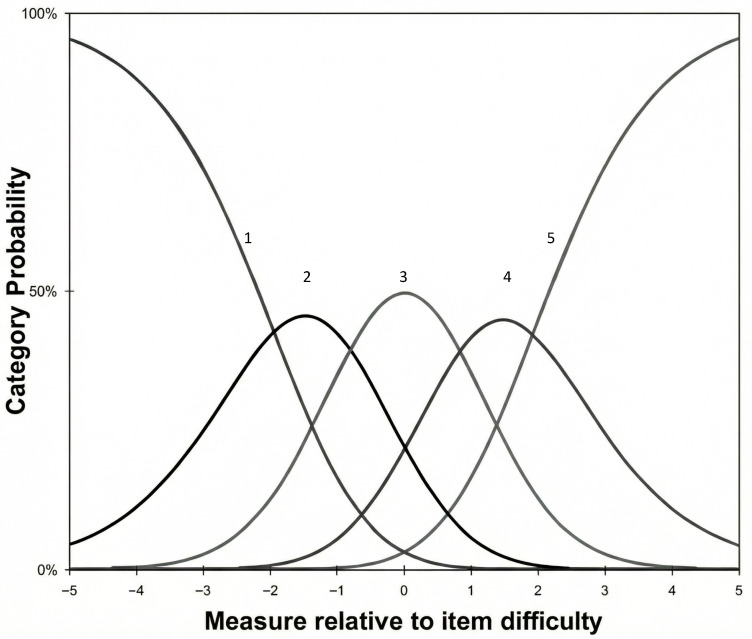
Category probability curves. Note. The curves represent the probability of endorsing each response category. The numbers above the curves (1–5) correspond to the response options on the 5-point Likert-type scale.

As can be seen in Figure 2, all the response options have, at some point along the continuum, a higher probability of being selected compared to the rest of the categories. Likewise, it is also observed that the threshold parameters of the categories increased monotonically (*τ*_1_ < *τ*_2_ < *τ*_3_ *< τ*_4_), that is, the threshold parameters corresponding to the intersection of the categories maintain a coherent order with the meaning of the response options (τ_1_ = −1.94, τ_2_ = −0.81, τ_3_ = 0.83, and τ_4_ = 1.92).

**Figure 3:**
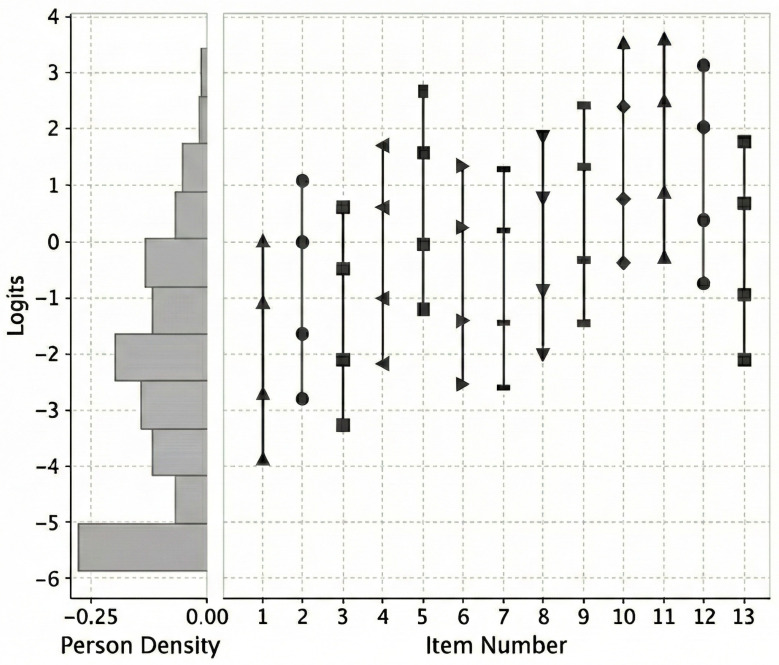
Item-person map.

Figure 3 shows the item-person map. On the left, the distribution of persons along the continuum of psychological pain is shown. At the top are the persons with the highest degree of psychological pain and at the bottom are those with the lowest degree of psychological pain (in logits). The persons (mean = −1.75 logits) are placed at a lower level of PS continuum than items (mean = 0.00 logits). Also, the distribution of persons’ logit scores, together with their equivalent in raw total scores, is shown in Table 3. As in Figure 3, in this table, we observe how the distribution is quite asymmetric, with the highest percentages of people in the low scores (floor-ceiling effect). On the right side of the item-person map, the items are located according to their “weight” (in logits) on the psychological pain continuum. In each of the items, four points are observed according to the thresholds of the response options. The distance between these thresholds is identical in each of the items, varying only their position on the continuum. As can be seen, the items cover an approximate range of between −4 and 4 logits of the psychological pain continuum. In terms of item content, items 10 (*No puedo soportar más mi dolor* [*I can’t take my pain anymore*]), 11 (*A causa de mi dolor, mi situación es inaguantable* [*Because of my pain, my situation is impossible*]), and 12 (*Mi dolor me está haciendo pedazos* [*My pain is making me fall apart*]) were placed in the high range of the psychological pain continuum. In contrast, items 1 (*Siento dolor psicológico* [*I feel psychological pain*]), 3 (*Mi dolor psicológico parece peor que cualquier otro dolor físico* [*My psychological pain seems worse than any physical pain*]), and 2 (*Siento un dolor interno* [*I seem to ache inside*]) were placed in the lower range of the continuum. Hence, these items require the least amount of psychological pain severity to score with response options that show a higher frequency or intensity, or both. Items 4, 8, and 13; items 6 and 7; and items 10 and 11 have small differences in item severities. An interitem separation of <0.15 logits indicate redundancy of the items, i.e., indicating overlap between these item difficulties [55].

Finally, the DIF study (Table 4) shows that no item presents notable differences between the subgroup of women and the subgroup of men. However, item 6 (*No entiendo por qué sufro* [*I can’t understand why I suffer*]) was statistically significant in the Rasch–Welch *t*-test (*p* = 0.012), suggesting some caution about a potential bias against women compared to men on this item.

## 4. Discussion

To our knowledge, the present study is the first to analyze the scores of the Spanish version of the PS [21] using a polytomous Rasch rating scale model [30]. In general, as a quality control principle, the analysis of the fit of the data to the model showed that the values of the items and individuals are in the established range to be considered adequate to the requirements of the Rasch model and productive for measurement [56]. However, item 6 presents a slight misfit (i.e., underfit), which means that there is more randomness (i.e., noise) in the data of this item with respect to the model, so these data are less predictable than the model expects, implying more ambiguity [50]. In case we were involved in a new measure development, item 6 could be considered unproductive for the construction of the measure (although not degrading) and could be replaced. However, we are analyzing data from an existing test, so it seems appropriate to maintain item 6 and evaluate in future analyses if the fit values are misfitting [50,57].

Regarding the separation indices and strata, the results obtained indicate that the scores of the Spanish version of the PS distinguished three strata of psychological pain for persons. In the case of the item, the findings show that the item is adequately distributed along the continuum and discriminates between many strata or levels of psychological pain [58].

Regarding the study of the response categories, the curves showed that each category had a region of the PS continuum in which they had a higher probability of being selected. Likewise, the thresholds showed an adequate order, with an orderly increase and in line with the meaning of the categories. In addition, the magnitudes of the distances between adjacent threshold estimates should indicate a noticeable step on the psychological pain continuum to show an empirical distinction between categories. In this case, the distances between adjacent thresholds were found to be neither too close nor too far apart, and close to the recommended optimal value of 1.4 logits [36,59,60]. In short, the study of the categories revealed relevant characteristics that support the use of 5-point Likert-type response options in the Spanish version of the PS.

Regarding the distribution of persons observed in the item-person map, persons were mostly located in the lower part of the psychological pain continuum. If we consider the ascertained relationship between psychological pain and suicidal behaviors [3,5], it seems reasonable to expect a distribution of psychological pain scores like the one obtained in this sample, as most of the young adults who participated did not manifest suicidal ideation or other suicidal behaviors. As for the distribution of the items, it stands out that items 10, 11, and 12 were the ones positioned at the highest part of the continuum. According to Shneidman [9], the unbearability or intolerability of psychological pain is the characteristic of this experience most associated with suicide. However, the PS items measure general experiences of psychological pain, considering the construct broadly and without focusing on a particular degree of bearability. Therefore, to design a brief measure of unbearable psychological pain, Pachkowski et al. [61] identified PS items 10, 11, and 12 as referring to greater unbearability of psychological pain. Although the brief measure of unbearable psychological pain (comprising these three items) did not significantly contribute to the improved prediction of suicide attempts compared to the complete PS [62], the placement of these items at the high end of the continuum supports the consideration of unbearable or intolerable psychological pain as the most extreme form of this experience, deserving of special clinical attention.

Finally, the differential item functioning (DIF) study showed an apparent bias against women compared to men in item 6 of the Spanish version of the PS (“I can’t understand why I suffer”). However, the size of the DIF was not large enough in magnitude to be considered relevant [51]. In short, all items of the Spanish version of the PS are free of a potential bias that would adulterate the interpretation of the scores according to the sex of the participants, although new empirical evidence would be able to corroborate whether the slight differential functioning of item 6 prevails.

However, these findings must be weighed against the sampling strategy. Despite the statistical robustness provided by a large sample and demographic quotas, the reliance on non-probabilistic recruitment via digital networks may introduce a potential selection bias. This suggests that generalizations to the broader population should be made with appropriate caution.

## 5. Conclusions

In conclusion, the present study provides new evidence that supports the use of the Spanish version of the PS to measure psychological pain. Beyond traditional findings based on CTT, the Rasch analysis reveals a severity-based hierarchy of the items that improves the interpretation of the scores. Specifically, high ratings on the items located at the upper end of this continuum serve as a “red flag” for unbearable pain [61], a critical state identified as a proximal precursor to suicide [7]. Consequently, the PS constitutes a valuable resource for psychological assessment and prevention strategies, providing a sensitive metric to detect distress deserving of attention [6] and to monitor suicide risk fluctuations independently of specific psychopathological diagnoses [2].

## Figures and Tables

**Table 1:** Socio-demographic characteristics of participants.

		*N* (%)
**Sex**	Woman	463 (51.2%)
	Man	440 (48.6%)
	Other	2 (0.2%)
**Completed education level**	No studies	19 (2.1%)
	Primary education	28 (3.1%)
	Secondary education	532 (58.8%)
	Higher education	326 (36.0%)
**Marital status**	Single	509 (56.4%)
	Married/Domestic partner/Stable partner	370 (41.0%)
	Divorced	23 (2.5%)
	Widowed	1 (0.1%)
**Work activity**	Employee	434 (48.0%)
	Student	390 (43.1%)
	Unemployed	59 (6.5%)
	Homemaker	21 (2.3%)

**Table 2:** Item measures, mean square fit statistics, item-total correlations, and threshold parameters.

Items	Measure	Error	INFIT		OUTFIT		Item-Total Correlation
			MNSQ	ZEMP	MNSQ	ZEMP	
Person M	−2.70	0.82	1.00	−0.10	0.95	−0.10	
Person SD	2.28	0.59	0.59	1.00	0.60	1.00	
Item M	0.00	0.06	1.02	0.00	0.95	−0.20	
Item SD	0.52	0.01	0.28	1.00	0.31	1.00	
1. Siento dolor psicológico. I feel psychological pain	−0.95	0.05	0.77	−1.02	0.88	−0.52	0.86
2. Siento un dolor interno. I seem to ache inside.	−0.42	0.05	0.85	−0.63	0.93	−0.27	0.82
3. Mi dolor psicológico parece peor que cualquier otro dolor físico. My psychological pain seems worse than any physical pain.	−0.65	0.05	1.30	1.10	1.16	0.64	0.79
4. Mi dolor hace que me den ganas de gritar. My pain makes me want to scream.	−0.10	0.06	1.37	1.28	1.35	1.11	0.74
5. Mi dolor hace que mi vida sea oscura. My pain makes my life seem dark.	0.37	0.06	0.82	−0.68	0.69	−1.06	0.77
6. No entiendo por qué sufro. I can’t understand why I suffer.	−0.29	0.05	1.72	2.32	1.79	2.43	0.70
7. Psicológicamente, me siento fatal. Psychologically, I feel terrible.	−0.32	0.05	0.66	−1.51	0.75	−1.05	0.83
8. Me duele porque me siento vacío/a. I hurt because I feel empty.	−0.03	0.06	1.01	0.04	0.95	−0.17	0.77
9. Me duele el alma. My soul aches.	0.24	0.06	0.98	−0.06	0.85	−0.48	0.76
10. No puedo soportar más mi dolor. I can’t take my pain anymore.	0.78	0.06	0.90	−0.35	0.74	−0.73	0.72
11. A causa de mi dolor, mi situación es inaguantable. Because of my pain, my situation is impossible.	0.83	0.06	0.87	−0.46	0.66	−0.96	0.72
12. Mi dolor me está haciendo pedazos. My pain is making me fall apart.	0.59	0.06	0.89	−0.41	0.66	−1.05	0.74
13. Mi dolor psicológico afecta a todo lo que hago. My psychological pain affects everything I do.	−0.07	0.06	1.08	0.29	0.94	−0.22	0.78
Threshold parameters		*τ*_1_ = −1.94, *τ*_2_ = −0.81, *τ*_3_ = 0.83, *τ*_4_ = 1.92					

**Table 3:** Raw total scores, estimated PS scores (logit units), and frequencies of scores in the sample.

PS Estimate	Raw Score	Mean SE	Frequency	% of the Sample
−5.83/−3.10	13–17	1.34	388	42.87%
−2.83/−2.07	18–22	0.44	150	16.57%
−1.92/−1.37	23–27	0.37	115	12.71%
−1.25/−0.78	28–32	0.34	59	6.55%
−0.66/−0.21	33–37	0.34	70	7.73%
−0.10/0.34	38–42	0.33	41	4.53%
0.45/0.90	43–47	0.34	31	3.43%
1.02/1.50	48–52	0.35	25	2.76%
1.63/2.23	53–57	0.38	18	1.99%
2.40/3.41	58–62	0.50	8	0.88%

**Table 4:** Different item functioning (DIF).

Items	DIF Measure (Women)	DIF Measure (Men)	DIF Contrast	Rasch–Welch *t*-Test (*p*)
Item 1	−0.95	−0.95	0.00	1.000
Item 2	−0.44	−0.38	−0.06	0.593
Item 3	−0.69	−0.60	−0.09	0.396
Item 4	−0.19	0.02	−0.21	0.058
Item 5	0.42	0.32	0.10	0.422
Item 6	−0.17	−0.44	0.27	0.012
Item 7	−0.39	−0.22	−0.16	0.133
Item 8	0.06	−0.14	0.20	0.075
Item 9	0.16	0.36	−0.20	0.088
Item 10	0.78	0.78	0.00	1.000
Item 11	0.86	0.81	0.05	0.713
Item 12	0.67	0.51	0.16	0.190
Item 13	−0.07	−0.05	−0.02	0.852

## Data Availability

The data are available to any person upon request to the e-mail address of the correspondence author.
